# Impact of In Vitro Gastrointestinal Digestion on the Phenolic Bioaccessibility and Bioactive Properties of Insect-Containing Beef Burgers

**DOI:** 10.3390/antiox13030365

**Published:** 2024-03-18

**Authors:** Gabriele Rocchetti, Gokhan Zengin, Gianluca Giuberti, Mariasole Cervini, Luigi Lucini

**Affiliations:** 1 Department of Animal Science, Food and Nutrition, Università Cattolica del Sacro Cuore, Via Emilia Parmense 84, 29122 Piacenza, Italy; 2 Department of Biology, Science Faculty, Selcuk University, Konya 42130, Turkey; gokhanzengin@selcuk.edu.tr; 3 Department for Sustainable Food Process, Università Cattolica del Sacro Cuore, Via Emilia Parmense 84, 29122 Piacenza, Italy; gianluca.giuberti@unicatt.it (G.G.); mariasole.cervini@unicatt.it (M.C.); luigi.lucini@unicatt.it (L.L.)

**Keywords:** novel foods, house cricket, migratory locust, mealworm, phenolic compounds, antioxidant properties, UHPLC-HRMS

## Abstract

Mealworm, migratory locust, and house cricket have recently been recognized by the European Commission as novel foods, thus being suitable in different food applications. In this work, we tested their powders as meat extenders at 5% (*w*/*w*) inclusion in beef burgers, considering their ability to vehicle phenolic compounds during simulated in vitro static gastrointestinal digestion (INFOGEST). Insect powders were abundant in different phenolic classes, recording the highest values in locust (LP; 314.69 mg/kg), followed by cricket (CP; 113.3 mg/kg) and mealworm (MWP; 51.9 mg/kg). Following a pan-cooking process, LP burgers were confirmed as the best source of phenolics, with a marked abundance of flavonoids and phenolic acids. Interestingly, the insect powders were found to affect the in vitro gastrointestinal bioaccessibility of phenolic compounds when compared with the CTR burger, likely promoted by the interactions between the phenolic compounds and proteins characterizing the tested insect powders. Among the most discriminant phenolic metabolites at the gastrointestinal level, we found several phenolic acids (mainly hydroxycinnamics), recording the highest content for the digested CP-containing burgers. Finally, stilbenes showed significant correlation values at the intestinal level with both antioxidant and enzymatic activities, while total flavonoids were the most correlated with the inhibition of acetylcholinesterase. Taken together, our preliminary findings demonstrated that insect powders added to beef burgers can promote the bioaccessibility and potential bioavailability of phenolics in the distal tracts of the intestine.

## 1. Introduction

To date, the European Food Safety Authority (EFSA) has authorized four edible insects as novel foods, as detailed in the Regulation (EU) 2015/2283. Among these, it is possible to list the following: ‘*Tenebrio molitor*’ larva (mealworm, as frozen, dried, and powder forms), ‘*Locusta migratoria*’ (migratory locust, grasshopper), ‘*Alphitobius diaperinus*’ larva (frozen, freeze-dried formulations), and ‘*Acheta domesticus*’ (dried, ground, frozen, and partially defatted whole house cricket) [1]. Therefore, considering that trading of these new products (in different forms) is regulated by Regulation (EU) No. 2017/2470, it is important to evaluate both the chemical and technological aspects of this new novel foods category [2]. 

In this scenario, edible insects represent one of the major future foods [3,4]. Particularly, edible insects are reported as a rich source of proteins, fats, fiber, vitamins, and minerals together with large amounts of polyphenols acting as bioactive compounds [5]. Among the edible insects, cricket (*A. domesticus)* is reported to be a rich source of phenolic acids, such as 4-hydroxybenzoic acid, *p*-coumaric acid, ferulic acid, and syringic acid, while mealworm (*T. molitor*) has been outlined as a source of bioactive antioxidant compounds, such as flavonoids and carotenoids [6]. Interestingly, no comprehensive reports are available to date regarding the phenolic composition of locust (*L. migratoria*) and locust-based ingredients. It is important to mention that the nutritional value of edible insects is highly variable [2] and their chemical composition is generally greatly variable even within the same species. In this regard, the species of the order *Orthoptera*, including crickets, grasshoppers, and locusts, is reported to show variations in the total protein content from 6 up to 77% [2]. According to the existing scientific literature, this variation is not only associated with different species and developmental stages, but it is also dependent on different types of feeding systems and geographical origins, as well as differences in the analytical methods used for protein determination [2,7].

As reviewed by Borges et al. [8], several studies are available to date dealing with the incorporation of edible insects into different food products. A huge amount of studies are based on the addition of insect-based ingredients to bakery products to increase their overall nutritional value. The second research line deals with the reformulation of meat products and the reduction/replacement of meat proteins. As far as bakery products are concerned, several research reports are available regarding their enrichment with insect powders (mainly from cricket and mealworm) [9]. Other studies also investigated in vitro the antioxidant potential of bakery products added with insects following a gastrointestinal digestion in comparison with an insect-free control product, revealing that insects are potentially able to vehicle bioaccessible antioxidant phenolics at the gastrointestinal level [2,10]. However, when the meat science area is targeted, no similar studies can be detected in the scientific literature, thus opening the way towards the enrichment of meat products with insect powders to study the phenolic bioaccessibility during an in vitro simulated digestion process. 

In a recent research work from our research group [1], we added 5% (*w*/*w*) of different commercial insect powders (authorized as novel foods) in beef meat to formulate insect-containing beef burgers to comprehensively evaluate the effect of insect powder incorporation on the textural, sensory, and cooking properties, also inspecting those bioactive peptides potentially accounting for enzyme inhibition during simulated in vitro gastrointestinal digestion. Therefore, starting from these background conditions, we carried out a comprehensive and never-before reported investigation dealing with the incorporation of insect powders in beef burgers that were cooked and in vitro digested following the static and harmonized INFOGEST protocol. Our aim was to provide new insights into the impact of the cooking process on the phenolic profile of insect-containing beef burgers, thus evaluating in vitro the bioaccessibility of the main phenolic classes in each post-digestion phase. 

## 2. Materials and Methods

### 2.1. Insect Powders and Manufacturing of Beef Burgers

Insect powders analyzed and used as ingredients were of commercial origin. Briefly, a cricket (*Acheta domesticus*) powder (CP) was obtained from boiled and dehydrated crickets, while a locust (*Locusta migratoria*) powder (LP) was obtained from microwave-dried and milled locusts. CP and LP were supplied by JR Unique Foods (Udon Thani, Thailand). On the other hand, a dried yellow mealworm (*Tenebrio molitor*) powder (MWP) was derived from oven-roasted mealworms and supplied by Bug Bazaar (Helsinki, Finland). More information regarding the origin and quality of the same commercial insect powders tested can be found in Rocchetti et al. [1]. 

The manufacturing process of insect-containing beef burgers was carried out by the meat pilot plant at the Università Cattolica del Sacro Cuore (Cremona, Italy), starting from four kg of three independent meat batters (beef meat 98.8% obtained from silver-side and top-side cuts, 5% fat, 1% of NaCl, and 0.1% of ascorbic acid). The resulting mixture was divided into four equal batches, namely a control batch (CTR burgers, where no insect powder was added), and three batches containing 5% (*w*/*w*) of each insect powder, namely MWP burgers, LP burgers, and CP burgers. More details regarding the preparation of the same burgers are reported in [1]. Basically, the manual burgers were shaped into portions of 100 g in weight and 100 mm in diameter. Taken together, three biological replicates (n = 3) for each burger type were considered. 

### 2.2. In Vitro Gastrointestinal Digestion of Cooked Burgers Added with Insect Powders

Prior to the in vitro digestion, samples were cooked in a preheated frying pan. Again, the full, accurate details regarding the cooking process adopted are comprehensively reported in Rocchetti et al. [1]. Briefly, the cooking process was performed in a preheated pan to standardize the cooking conditions (6 min at a hotplate power of 1400 W, with one turn at 3 min, thus reaching an ideal internal temperature of 72 ± 1 °C). 

The INFOGEST harmonized in vitro static gastrointestinal digestion method detailed by Brodkorb et al. [11] was then used. This protocol is based on three consecutive in vitro digestion phases, namely oral (2 min, 37 °C, pH 7.0), gastric (120 min, 37 °C, pH 3.0), and intestinal (120 min, 37 °C, pH 7.0). All the in vitro conditions and the specific concentration of each enzyme involved in the in vitro digestion protocol were detailed and justified by Minekus et al. [12] and by Brodkorb et al. [11]. Additionally, a rabbit gastric extract that contained both gastric lipase and pepsin was used during the gastric phase [11,13]. All the in vitro conditions and buffers used were scaled up considering 50 g for each cooked burger sample. Additionally, an automatic meat grinder [1] was used to mimic the mastication process. Liquid aliquots (n = 3, biological replicates, representing the bioaccessible fraction) were collected at the end of each in vitro static digestion phase and stored at −18 °C until further analysis.

### 2.3. Extraction and UHPLC-HRMS Untargeted Phenolic Profiling

The extraction of phenolic compounds from raw insect powders and insect-containing cooked burgers was performed by weighting 2 g of each material in 20 mL of a hydroalcoholic solution (80% Methanol *v*/*v*) acidified with 0.1% formic acid [14]. The extraction was promoted by a homogenizer-assisted extraction system using an Ultraturrax (Ika T25, Staufen, Germany) for 3 min at maximum speed. Thereafter, extracted samples were centrifuged for 15 min at 5500 rpm (4 °C) and the supernatants were filtered (0.2 micron syringe filters) in UHPLC vials until further analyses. Additionally, as far as the aliquots collected from in vitro gastrointestinal digestion are concerned, they were firstly centrifuged (15 min, 5500 rpm at 4 °C) and then the supernatants were filtered (0.2 micron syringe filters) in UHPLC vials until further analyses.

The untargeted phenolic profiling was carried out using a high-resolution mass spectrometry (HRMS) approach. A Q-Exactive™ Focus Hybrid Quadrupole-Orbitrap Mass Spectrometer (Thermo Scientific, Waltham, MA, USA) was used. The full analytical details regarding both chromatography and HRMS can be found in Rocchetti et al. [1]. We used a full scan MS data-dependent (Top N = 3) MS/MS mode to acquire ions in the mass range 80–1200 *m*/*z*, using a positive ionization mode. The resolution of the HRMS consisted of 70,000 FWHM (full scan) and 17,500 FWHM (data-dependent MS/MS mode), considering the collision energies 10, 20, and 40 eV. The heated electrospray ionization (HESI) parameters are described in a previous work [15]. The generated raw data were then processed and statistically analyzed using the software MS-DIAL (version 4.90) [16], considering a level 2 of confidence in annotation [17]. This was achieved through spectral matching against the comprehensive databases FooDB and Phenol-Explorer, setting a mass accuracy condition < 5 ppm. 

### 2.4. Bioaccessibility of Phenolic Compounds Following In Vitro Gastrointestinal Digestion

The different polyphenols annotated were classified according to the information available on the Phenol-Explorer database and then the cumulative intensities were converted to mg phenolic equivalents kg^−1^ dry matter (DM) by means of calibration curves of pure standard compounds (Extrasynthese, Lyon, France). The standards used were sesamin (lignans), ferulic acid (phenolic acids), cyanidin (anthocyanins), oleuropein (tyrosols and low-molecular-weight phenolics), resveratrol (stilbenes), catechin (flavan-3-ols), quercetin (flavonols), and luteolin (flavones and other remaining flavonoids). For calibration purposes, we considered a linear fitting (R^2^ > 0.98) and a calibration range of 0.5–500 mg·L^−1^. The % of bioaccessibility of the different phenolic subclasses during the three in vitro static gastrointestinal digestion phases was calculated as follows: 

% of Bioaccessibility: total content per class (mg equivalents kg^−1^) of digested burger samples/total content per class (mg equivalents kg^−1^) of insect-added cooked burger (starting material).

### 2.5. In Vitro Antioxidant and Enzymatic Inhibition Activities

The evaluation of the antioxidant properties during the in vitro gastrointestinal digestion of cooked insect-fortified beef burgers took place. This analysis encompassed the examination of DPPH• radical scavenging, ABTS•+ radical scavenging, cupric ion-reducing antioxidant capacity (CUPRAC), ferric ion-reducing antioxidant power (FRAP), and metal chelating activity. Additionally, the total antioxidant activity was quantified utilizing the phosphomolybdenum method. Their details were reported in a previous work [18]. The in vitro activity findings were expressed using standard units, specifically milligrams of trolox equivalents (TEs) per milliliter for the DPPH•, ABTS•+, CUPRAC, and FRAP assays, and milligrams of EDTA equivalents (EDTAEs) per milliliter for the metal chelating assay. The phosphomolybdenum assay results were quantified in millimoles of TE per milliliter.

Moreover, Uysal et al. [18] comprehensively documented the assessment of AChE, BChE, tyrosinase, α-amylase, and α-glucosidase inhibition, providing detailed experimental procedures for each. The activity data were standardized and expressed as milligrams of galantamine equivalents (GALAEs) per milliliter in the AChE and BChE assays, milligrams of kojic acid equivalents (KAEs) per milliliter in the tyrosinase assay, and millimoles of acarbose equivalents (ACAEs) per milliliter in the α-amylase and α-glucosidase assays.

### 2.6. Statistical Analysis

One-way analysis of variance (ANOVA; *p* < 0.05) was performed using the PASW Statistics 26.0 software (SPSS Inc., Chicago, IL, USA). Duncan’s post hoc test allowed for identifying homogenous subclasses when considering the phenolic profiles of the tested samples. A multivariate statistical analysis was performed using the online-available software MetaboAnalyst 5.0 and the licensed software SIMCA (Version 16; from Umetrics, Malmo, Sweden), considering both an unsupervised hierarchical clustering approach and a supervised orthogonal projection to latent structures discriminant analysis (OPLS-DA). The OPLS-DA model allowed for extrapolating the discriminant biomarkers of each post-digestion phase through the utilization of a VIP (variables’ importance in projection) selection method, considering those metabolites with a minimum prediction score value higher than 1. 

## 3. Results and Discussion

### 3.1. Phenolic Profiling of Insect Powders and Insect-Added Cooked Burgers

Concerning the raw insect powders, the untargeted metabolomics approach based on UHPLC-Orbitrap-ddMS^2^ mass spectrometry allowed for the putative identification of 16 anthocyanins, 10 flavan-3-ols, 18 flavonols, 39 flavones and other flavonoids, 60 other phenolics (i.e., lignans, alkylphenols, tyrosols, coumarins, and others), 60 phenolic acids (mainly hydroxycinnamics and hydroxybenzoics), and 7 stilbenes. All the information related to phenolic annotation (i.e., adduct type, total identification score, MS1 isotopic profiles, and MSMS spectra) is provided in Table S1. The semi-quantification values for the different phenolic classes are reported in Table 1. Overall, the LP was outlined as the best source of total phenolics (314.7 mg/kg), followed by the CP (113.3 mg/kg) and MWP (51.9 mg/kg). Looking at the different phenolic classes separately, we found a higher cumulative abundance of phenolic acids in each insect powder analyzed, with a great abundance of two hydroxybenzoic acids, namely gallic and hydroxycaffeic acids (Table S1). However, the most exclusive phenolic acid profile was recorded in the LP, revealing the presence of several compounds, such as (among others) ellagic acid, ellagic acid arabinoside, vanillic acid, and dihydroferulic acid 4-*O*-glucuronide. Regarding the distribution of flavonoids, the LP was the best source, followed by the MWP and CP, recording cumulative values of 31.8, 13.8, and 9.2 mg/kg, respectively. Interestingly, the MWP was outlined as the best source of flavan-3-ols and stilbenes, with 3′-*O*-methylepicatechin and piceatannol being the most abundant compounds (Table S1). 

As reviewed by Torres-Castillo et al. [4], the presence of phenolics may vary according to the diet and nutritional status of the insects. Although few investigations are available to date, the presence of polyphenols in insects was associated with their feeding regimen and with the ability of insects to synthesize polyphenols through the sclerotization process [2]. The presence of these compounds in edible insects resulted in them being highly relevant for human nutrition and food technology, especially because they can add further value and usefulness from the perspective of the food industry. Particularly, recent studies revealed that edible insects can provide bioactive phenolic compounds (mainly flavonoids) [19,20,21,22,23,24], owning different health-promoting properties, including the antioxidant, anti-inflammatory, anticancer, antimicrobial, and antibacterial inhibition of the pancreatic lipase enzyme, insulin regulation, and glycemic inhibition [2]. For instance, experimental in vitro studies demonstrated the antioxidant effect of polyphenolic compounds derived from the extracts of house crickets (*A. domesticus*), mealworms (*T. molitor*) [25], and dark black chafer beetles (*Holotrichia parallela*) [21]. 

Looking at the available literature reports, the most common polyphenolic compounds found in edible insects are represented by phenolic acids (such as gallic acid, 4-hydroxybenzoic acid, syringic acid, *p*-coumaric acid, caffeic acid, ferulic acid, and sinapic acid) and flavonoids (such as luteolin, apigenin, isorhamnetin, quercetin, kaempferol, vitexin, orientin, and their glycosidic forms) [4]. These compounds and their glycosidic derivatives have also been annotated in the MWP, CP, and LP under investigation, thus confirming a strong agreement with the already available literature reports. A comprehensive understanding of the phenolic profile of these insect raw ingredients can provide indications of their possible use as functional foods. However, it is also important to mention that insects are typically processed by roasting, freezing, extrusion, and blanching, and to date, no sufficient evidence evaluating the global effects of such processing methods on insects is yet available. In particular, while actual knowledge could suggest that mild processing can retain the bioactive potential and functionality of these phytochemicals, it is strongly advisable that the processing time and temperatures should be better evaluated and optimized to avoid significant losses [2].

As the next step, we evaluated the phenolic profile of cooked insect-containing burgers before undergoing the in vitro gastrointestinal digestion. Indeed, the cooked burgers were considered the starting material to calculate the phenolic bioaccessibility during the simulated consumption, considering that this product category must be cooked before consumption. The cumulative phenolic profile of the cooked burgers following the addition of 5% (*w*/*w*) of the insect powders tested is reported in Table S1. To summarize, the LP burgers were characterized by the highest cumulative phenolic content (131.9 mg/kg), followed by the CP and MWP burgers (being 65.6 and 56.7, respectively). The CTR sample (i.e., beef meat with no insect powder addition) showed a total phenolic content of 44.7 mg/kg, being particularly abundant in phenolic metabolites belonging to the subclasses of phenolic acids (both hydroxybenzoics and hydroxycinnamics) and other phenolics (Table S1), likely related to the animal diet and feeding conditions [26]. Looking at the semi-quantitative contents (Table S1), the LP provided the highest inclusion of phenolic acids, with a marked abundance of some compounds, mainly belonging to hydroxybenzoic acids (Table S1). Also, according to the phenolic profile reported in Table 1 for raw insect powders, we found that the MWP burgers were characterized by the highest contents of flavan-3-ols (2.0 mg/kg) and stilbenes (5.50 mg/kg), while the LP and CP burgers were the best source of flavones and other flavonoids, recording semi-quantitative values of 2.8 and 2.0 mg/kg, respectively (Table S1). As far as the subclass of flavonols is concerned, the LP burgers were outlined as the best source, recording a great abundance of quercetin 3-*O*-xyloside, clearly deriving from the insect powder and then representing a biomarker of the inclusion of LP in the beef meat under investigation. The presence of glycosidic forms of quercetin has also been reported in Carolina locust (*Dissoteira carolina*) as resulting from the absorption of quercetin glycosides from the plant tissues [27]. Overall, our data on the phenolic composition of insect-added cooked burgers are difficult to compare with the existing literature; in this regard, the majority of the published works are based only on the evaluation of the technological properties of the formulated meat products (such as texture parameters, color, and cooking properties) [28,29]. For example, Cavalheiro et al. [29] recently tested the potential of cricket (*A. domesticus*) flour as a lean meat replacer in the development of beef patties, demonstrating that the incorporation of up to 5.0% (*w*/*w*) of cricket flour into beef patties was optimal in terms of the composition, technological, sensorial, and cooking properties. 

### 3.2. Multivariate Statistical Discrimination of Insect-Added Cooked and Digested Burgers and Phenolic Bioaccessibility

**Figure 1:**
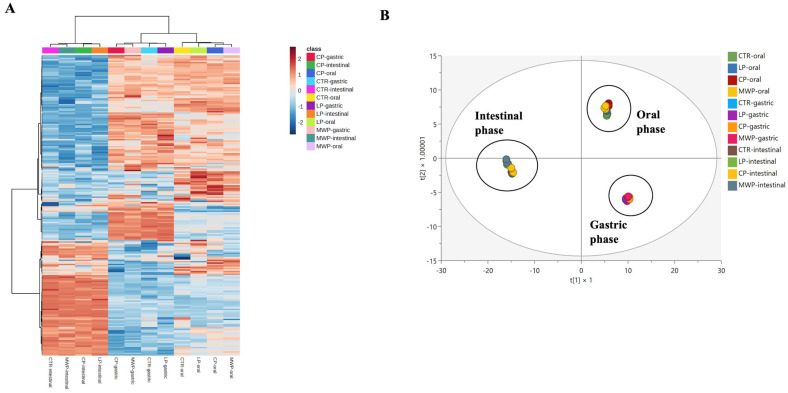
Averaged unsupervised hierarchical clustering heat map (**A**) built considering the log_2_ fold-change distribution of each metabolite in the different sample groups, together with (**B**) orthogonal projection to latent structures discriminant analysis (OPLS-DA) score plot built considering the polyphenolic composition of insect-added burgers vs. the control sample, moving from post-oral to post-gastric and pancreatic phases of in vitro simulated gastrointestinal digestion.

Once the phenolic profiles of the cooked burgers were monitored, the INFOGEST protocol was used to evaluate the main changes in these compounds following the oral (2 min), gastric (120 min), and intestinal digestion (120 min) phases. Firstly, we carried out both an unsupervised and supervised statistical approach to check for similarities and differences in the chemical profiles measured in the different phases. The hierarchical clustering heat map (based on monitoring the log_2_ fold-change variations in each metabolite detected) was used as an unsupervised strategy to evaluate the modifications of phenolics during in vitro gastrointestinal digestion. As shown in Figure 1A, two main clusters could be outlined; on the right side, a sample grouping was noticed including both the oral and gastric phases, while on the left side, an exclusive grouping including the intestinal phase could be noticed, thus showing clear modifications of the phenolic profile of the in vitro digested cooked burgers. Interestingly, looking at specific sub-clusters at the intestinal level (Figure 1A), it was possible to notice a chemical similarity between the digested LP and CP burgers (i.e., those owning the highest cumulative phenolic content), while the MWP-containing burgers were sub-grouped with the CTR burgers. Additionally, it was clear from the hierarchical clustering heat map that some groups of phenolic metabolites were strictly and exclusively associated with only the gastric and pancreatic digestion phases. As the next step, we used a supervised approach based on OPLS-DA to predict the modifications of phenolics during in vitro gastrointestinal digestion, thus extrapolating the most discriminant metabolites of the trends observed. The orthogonal latent vector (Figure 1B) clearly separated the pancreatic phase from the other ones, thus corroborating what was outlined from the unsupervised heat map (Figure 1A). 

The OPLS-DA score plot distribution observed (Figure 1B) was mainly due to 27 phenolic metabolites that were extrapolated through a VIP selection method, considering a VIP score > 1 as a minimum threshold, classified and reported in Table 2, together with their VIP score, cross-validated standard error, and association with the insect powders under investigation. Phenolic acids were the most represented phenolic class among the discriminant biomarkers, including 13 compounds, with the phenolic metabolite 5-(3′,4′-dihydroxyphenyl)-valeric acid owning the highest VIP score (1.665) and being a biomarker for the consumption of CP-containing burgers (Table 2), followed by cinnamic acid (1.600), which was outlined as a biomarker of the digested LP-containing burgers. Interestingly, a high number of the discriminant phenolics were mostly abundant in the LP, thus confirming the potential of this insect powder to vehicle bioactive phenolics to the small intestine. As far as the discriminant biomarkers of the MWP are concerned, the most important ones were definitely dihydrocaffeic acid, caffeic acid 3-*O*-glucuronide, and the stilbene piceatannol. The VIP selection method allowed us to also record some meat phenolic metabolites (not detected in the raw insect powders), namely 3-caffeoylquinic acid (also known as chlorogenic acid), 5-(3′,5′-dihydroxyphenyl)-gamma-valerolactone 3-*O*-glucuronide, epicatechin 3-*O*-gallate, and gallagic acid (Table 2).

Therefore, both the unsupervised (hierarchical clustering approach) and supervised (OPLS-DA) findings revealed a huge modification of the phenolic profiles of the insect-containing burgers vs. the CTR. To better investigate these changes, the % of bioaccessibility was extrapolated considering each post-digestion phase separately, namely (post) oral, (post) gastric, and (post) pancreatic ones. The results obtained are summarized in Table 3, considering the seven phenolic classes targeted together with their semi-quantitative values and % of bioaccessibility vs. the cooked burgers (before in vitro digestion). Looking at the distribution of the different phenolic classes, we found a very low % of bioaccessibility of phenolics following the oral digestion phase, with the highest values recorded for anthocyanins in the CTR and LP-containing burgers, respectively, while the lowest % of bioaccessibility values were measured for flavones, being in the range 0.01–0.2% (Table 3). Overall, the low bioaccessibility values recorded during the oral digestion phase can be explained by the limited time of enzyme exposure (i.e., 120 s) and to the sole use of amylases; in this regard, as also highlighted by García-Pérez et al. [30], the conditions adopted may prevent the release and further solubilization of phenolic metabolites from the meat matrix. In addition, it is known that while the mechanical disruption of the meat structure enables their partial release and bioaccessibility in the gut, it also increases the surface area for possible interactions with digestive enzymes [31]. Also, the potential interactions between meat proteins and polyphenols (provided by insect addition) can significantly alter the polyphenols’ availability for digestion [31]. 

As far as the gastric phase is concerned, higher % of bioaccessibility values were measured for each phenolic subclass targeted; in particular, anthocyanins ranged between 4.1 and 27.6%, flavan-3-ols were in the range 2.3–5.6%, flavones in the range 0.1–1.8%, flavonols in the range 0.4–9.5%, phenolic acids in the range 2.5–10.4%, other phenolics in the range 4.9–7.3%, and stilbenes 0.3–2.1% (Table 3). Interestingly, although being generally more abundant in the cooked insect-containing burgers, several phenolic subclasses were less available for absorption following the gastric digestion phase when compared with the polyphenols from the CTR burgers. This consideration was particularly evident for anthocyanins, flavan-3-ols, phenolic acids, and stilbenes. On the other hand, the MWP burgers provided relatively higher bioaccessibility values for two flavonoid subclasses, namely flavone equivalents and flavonols. Therefore, the in vitro gastrointestinal behavior of the LP and CP cooked burgers seems to suggest that phenolic compounds from these two sources were scarcely hydrolyzed at the typical conditions of gastric digestion (i.e., pH 3.0), likely due to a combined effect promoted by the interactions between insect protein/peptides and phenolic compounds within the food matrix. Among the most abundant compounds characterizing the gastric digestion phase (Table S1), we found protocatechuic acid 4-*O*-glucoside (phenolic acids), catechin 3-*O*-gallate (flavan-3-ols), eriocitrin (flavone equivalents), glycosylated derivatives of kaempferol (flavonols), several lower-molecular-weight phenolics such as bergapten, psoralen, and esculetin (Table S1), and e-Viniferin (stilbene). The increase in the simple and lower-molecular-weight phenolic substances during in vitro digestion, such as protocatechuic acid derivatives and other phenolic acids, has already been described in the scientific literature and mainly justified considering a higher stability of these compounds due to their simple structures, and/or to their facilitated release from the food matrix during the simulated process. However, the potential decomposition and transformation of higher-molecular-weight compounds into simple phenolics during the in vitro digestion process cannot be excluded [32].

Finally, the % of bioaccessibility was evaluated following the 120 min incubation (37 °C, pH 7.0) step. As reported in Table 3, phenolic acids and flavonols were outlined as the most bioaccessible, and then available for absorption, phenolic subclasses; in particular, bioaccessibility values in the ranges 4.8–24.6% for flavonols and 11.4–35.4% for phenolic acids could be measured. Focusing on the impact of the 5% (*w*/*w*) insect addition, we again found lower % of bioaccessibility values for almost all phenolic subclasses when compared with the CTR burgers, except for flavonols in the MWP burgers (Table 3). Among the most abundant phenolic metabolites at the intestinal level, we found several derivatives of catechin and epicatechin (Table S1), glycosylated forms of chrysoeriol (Table S1), glucuronide- derivatives of flavones (Table S1), some lignans (highly and exclusively bioaccessible at the intestinal phase), together with typical phenolic metabolites resulting from the intestinal modification of phenolic acids and flavonoids (Table S1). The results obtained at the post-pancreatic level confirm the behavior previously hypothesized for the gastric phase; in particular, the inclusion of insect powders was found to reduce the intestinal bioaccessibility of the main phenolic classes characterizing the raw powders, thus suggesting the ability of insect powders to vehicle phenolic compounds to the distal tracts of the large intestine, where the undigested insect-added meat matrix could undergo a microbial fermentation and then contribute to the appearance of typical phenolic metabolites resulting from the processing of unmodified parent compounds [33]. This behavior is usually observed in fruit and vegetable matrixes, where the bioavailability of phenolic antioxidants is determined by their low bioaccessibility due to physical and chemical interactions between these compounds and the non-digestible polysaccharides of the cell walls [34]. Although requiring further works (using, for example, a representative fecal inoculum to simulate the colonic fermentation) based on ad hoc experimental conditions, these preliminary findings suggest that the insect powders, as a source of proteins and phenolic compounds, could potentially represent a good source at the large intestinal level of bioactive molecules owning different bioactive properties. 

In this regard, besides phenolic compounds, our previous work [1] revealed that the in vitro gastrointestinal digestion of the same insect-added burgers is also able to provide potentially bioactive peptides recognized to act as enzyme modulators. Following the incorporation of proteins and peptides as ingredients in food formulations, it is essential to consider their interactions with other compounds, which can lead to changes in bioactive properties [35]. In this regard, the non-covalent interactions between peptides and polyphenols through hydrogen bonds, ionic interactions, and hydrophobic–hydrophobic interactions can potentially modify their bioactive properties. Considering the use of insect proteins to obtain bioactive peptides and their application as ingredients in food formulations, evaluating phenolic compound–peptide interactions is gaining interest. However, this research area remains an emerging field and has yet to be explored extensively in the context of insect protein hydrolysates [35]; our experimental findings provide a first degree of knowledge on the potential impact of phenolic–peptide interaction on the bioaccessibility of these bioactive compounds. Interestingly, to the best of our knowledge, the fortification of complex food matrices, including meat, with phenolic extracts and other meat extender categories together with the evaluation of phenolic bioaccessibility has been scarcely studied to date. A previous study by Pesic et al. [36] demonstrated that the addition of a complex food matrix to a grape skin extract (as a source of polyphenols) did not significantly affect the recovery of the major grape skin compounds, but the total recovery of the major phenolic classes was significantly lower due to the contribution of the food matrix polyphenols to the total amount of phenolics in the mixture before digestion. Therefore, it seems crucial to carefully evaluate the significant contribution of the food matrix (meat) and digestive fluids to the final polyphenolic content and potential bioactivity detected. 

### 3.3. Antioxidant and Enzymatic Properties Following the Simulated Consumption of Insect-Added Cooked Burgers and Correlation Results

In recent years, insects have become increasingly important as ingredients in functional foods. In this sense, determining their antioxidant properties can provide important insights for further nutraceutical exploitation [37,38,39]. We examined the antioxidant properties of insect-containing beef burgers after in vitro gastrointestinal digestion and the results are shown in Table 4. The radical quenching abilities of the digested samples were tested by ABTS and DPPH assays, which are the most common among in vitro antioxidant assays. The best DPPH radical scavenging ability was observed in the LP-added burgers (6.94 mg TE/mL), while the MWP-added burgers were the most active in the ABTS scavenging assay (35.37 mg TE/mL). In the ABTS radical scavenging assay, the weakest ability was found in the CTR-fortified (30.59 mg TE/mL) and CP-fortified burgers (31.83 mg TE/mL) (*p* > 0.05). Furthermore, as part of the investigation into additional crucial mechanisms, reducing power assays were conducted. These assays, namely CUPRAC and FRAP, involve the reduction of Cu^2+^ to Cu^+^ and Fe^3+^ to Fe^2+^ through the action of antioxidants. They serve as measures of the electron-donating potential of antioxidants. As can be seen from Table 3, in both the FRAP and CUPRAC assays, the highest ability was found in the LP-added burgers (CUPRAC: 28.82 mg TE/mL and FRAP: 15.91 mg TE/mL), followed by the CP-, MWP-, and CTR-digested burger samples. The chelation of transition metals controls the production of hydroxyl radicals in the Fenton reaction. In contrast to radical scavenging and reducing power tests, the MWP-added burgers showed the strongest metal chelating ability (7.82 mg EDTAE/mL), but the value was quite similar to the CP-added burgers (7.39 mg EDTAE/mL). The phosphomolybdenum assay also involves the reduction of Mo (VI) to Mo(V) by antioxidant compounds. Since not only phenolics but also non-phenolic antioxidants (e.g., tocopherols, ascorbic acid, and carotenoids) can play a role in the test, it can be described as one of the total antioxidant tests. In addition, the assay has some limitations. For example, the assay performs incubation at 90 °C and possible protein precipitation could occur if the samples have a high protein content [40]. In this test, the three digested insect-containing burger samples (i.e., LP, MWP, and CP) showed a very similar ability, while the weakest value was found in the CTR burgers. When all the antioxidant results were evaluated together, the LP- and MWP-containing burgers showed stronger antioxidant properties than the CP and CTR burgers. The observed ability can be explained by the presence of some phenolic compounds acting as strong antioxidants. In this regard, as shown in Table 3, the highest phenolic content was detected in the LP-containing cooked burger samples [41,42]. Interestingly, the Pearson’s correlation analysis revealed that stilbenes were the class of phenolics mostly correlated with the antioxidant activities measured, recording significant correlation coefficients with DPPH (0.747; *p* < 0.01), CUPRAC (0.607; *p* < 0.05), and FRAP (0.737; *p* < 0.01). Several studies available in the scientific literature have shown that insect powders can have a positive effect on the preparation of functional foods. For example, in a previous study by Gaglio et al. [10], powdered larvae of mealworm (MW) and buffalo worm (BW) were selected to produce a functional bread, resulting in a significant increase in the antioxidant potential recorded following in vitro simulated gastrointestinal digestion. Additionally, Gumul et al. [43] reported that the inclusion of some insect flours (i.e., *Acheta domesticus*, *Alphitobius diaperinus*, and *Tenebrio molitor*) resulted in the increased antioxidant capacity of the prepared nut bars. A similar behavior was also reported by Kowalski et al. [44] for the preparation of cakes with insect flours (i.e., buffalo worm, cricket, and mealworm). Therefore, as a general consideration, insect ingredients (in the forms of powders and/or flours) can be considered as a source of natural antioxidants in the production of functional ingredients.

In the past decade, there has been a global rise in the prevalence of various diseases, underscoring the pressing need for immediate solutions to address this issue. Enzymes, recognized for their versatility, emerge as key players in disease management. According to the theory of enzyme inhibition, the reversible inhibition of essential enzymes can contribute significantly to alleviating the symptoms associated with the aforementioned diseases [45]. For example, acetylcholine (AChE) levels are lower in Alzheimer’s patients than in healthy people. If we reversibly inhibit acetylcholinesterase (AChE), the level can be increased and it could help improve cognitive function in these patients [46]. A similar fact can be observed between amylase/glucosidase and blood sugar level control in diabetics [47]. Although several compounds have been synthesized as enzyme inhibitors, most of them have adverse side effects. At this point, novel, effective, and safe enzyme inhibitors are important topics on the scientific platform. In the present study, we examined the enzyme inhibitory properties of insect-fortified samples after in vitro gastrointestinal simulation against cholinesterase, tyrosinase, amylase, and glucosidase. The results are shown in Table 5. In both the cholinesterase assays, the best ability was measured for the LP-digested cooked burgers (AChE: 0.35 mg GALAE/mL; BChE: 1.26 mg GALAE/mL), while the CTR burgers showed the weakest ability (AChE: 0.09 mg GALAE/mL; BChE: 0.56 mg GALAE/mL). As far as other bioactivities are concerned, we evaluated the inhibition potential against the enzyme tyrosinase. This is a key enzyme in the melanin synthesis and its inhibition is an important way to treat hyperpigmentation problems. The best tyrosinase inhibitory effect was observed in the MWP-containing burgers; however, the tested samples showed very similar tyrosinase inhibitory effects (12.65–13.51 mg KAE/mL). Similar to tyrosinase, the inhibitory effects of amylase and glucosidase were numerically very close in the samples tested. When the results obtained were jointly evaluated, the burgers containing LP showed a stronger inhibitory effect than the other samples. 

As an insight into the structure–ability relationship, some compounds might be attributed to the observed ability. In the correlation analysis, total flavonoids (as cumulative values of different flavonoid subclasses) showed a good correlation with the AChE inhibition observed, recording a significant (*p* < 0.05) correlation coefficient (0.586). Also, stilbenes were again strongly correlated with our in vitro findings, recording a significant correlation coefficient (0.767; *p* < 0.01). Consistently, several researchers have found stilbenes to be significant inhibitors [48,49] of intestinal enzymes, with some dimeric stilbenoids (such as viniferin isomers) described to be better than the reference drug acarbose in inhibiting the pancreatic α-amylase [50]. Coherently, our findings revealed a great abundance of stilbenoids at the intestinal level, such as e-viniferin and resveratrol derivatives, likely explaining the results obtained. To our knowledge, there is a scarcity of scientific literature on the enzyme inhibitory properties of insect powders and/or flours. However, some studies have highlighted the inhibitory effects of specific insects on dipeptidyl peptidase IV (DPP-IV) [51] and angiotensin-converting enzyme (ACE) [52]. Our results therefore suggested that the tested insect powders can be considered as functional ingredients in the preparation of health-promoting applications.

## 4. Conclusions

Edible insects, among the existing and recently authorized novel foods, represent a potential valuable source of bioactive compounds, such as polyphenols. This work, for the first time, provided evidence towards the exploitation of insect powders from mealworm, house cricket, and migratory locust in the formulation of enriched beef burgers, able to vehicle phenolic metabolites in the distal tracts of the intestine. The highest total phenolic content was measured in the locust powder, followed by the cricket and mealworm powders. Interestingly, we provided evidence that flavonoids and phenolic acids from insects are retained following a cooking process; however, low in vitro gastrointestinal bioaccessibility values of the different phenolic classes was measured when compared with the not-enriched beef burgers, likely promoted by the interaction network involving phenolic compounds and proteins/oligopeptides characterizing the tested insect powders. Phenolic acids (mainly hydroxycinnamics) were the most discriminant compounds during the in vitro gastrointestinal digestion step, being particularly abundant at the intestinal level in the CP-containing cooked and digested burgers. As far as the bioactive profile is concerned, stilbenes were the best correlated at the intestinal level with both antioxidant and enzymatic activities, while total flavonoids recorded a significant correlation with acetylcholinesterase inhibition. The limitations of this preliminary study deal with the utilization of an in vitro static gastrointestinal digestion to assess the phenolic bioaccessibility (based on untargeted metabolomics as annotation approach) and not the optimization of the insect powder percentage to be included in the beef burgers from a technological standpoint. Therefore, future perspectives are based on the in vitro fecal fermentation of the undigested burger fraction coupled with cellular in vitro bioavailability assays that are strongly required to evaluate how the colonic microbiota react to phenolic compounds provided by the insect powders under evaluation. 

## Figures and Tables

**Table 1:** Semi-quantitative content of the different phenolic class equivalents (Eq.) in the insect powders under investigation. Data are expressed as mean value (mg/kg; n = 3) ± standard deviation. Superscript letters within each row indicate significant differences as resulted from one-way ANOVA (*p* < 0.05) followed by Duncan’s post hoc test. Abbreviations: CP (cricket powder); LP (locust powder); MWP (mealworm powder).

Phenolic Subclass	CP	LP	MWP
Anthocyanins (Cyanidin Eq.)	0.05 ± 0.01 ^a^	2.61 ± 0.22 ^c^	0.68 ± 0.07 ^b^
Flavan-3-ols (Catechin Eq.)	2.14 ± 0.22 ^a^	7.35 ± 0.34 ^b^	10.67 ± 1.01 ^c^
Flavonols (Quercetin Eq.)	0.88 ± 0.16 ^a^	10.36 ± 1.04 ^b^	0.40 ± 0.04 ^a^
Flavones and other flavonoids (Luteolin Eq.)	6.17 ± 0.41 ^b^	11.49 ± 1.77 ^c^	2.01 ± 0.77 ^a^
Phenolic acids (Ferulic acid Eq.)	82.36 ± 4.91 ^b^	228.39 ± 9.79 ^c^	24.84 ± 1.31 ^a^
Other phenolics (Oleuropein Eq.)	20.11 ± 0.56 ^b^	49.40 ± 2.11 ^c^	6.01 ± 1.04 ^a^
Stilbenes (Resveratrol Eq.)	1.59 ± 0.08 ^a^	5.09 ± 0.69 ^b^	7.28 ± 0.40 ^c^
Total content Eq.	113.3	314.7	51.9

**Table 2:** VIP markers following supervised OPLS-DA during in vitro gastrointestinal digestion of different insect-containing burger samples.

Discriminant Compounds	Phenolic Subclass	VIP Score (OPLS-DA)	Most Abundant Source
5-(3′,4′-dihydroxyphenyl)-valeric acid	Hydroxyphenylpentanoics	1.665 ± 0.469	CP
Cinnamic acid	Hydroxycinnamics	1.600 ± 0.549	LP
Dihydrocaffeic acid 3-*O*-glucuronide	Hydroxyphenylpropanoics	1.392 ± 0.251	MWP
Scopoletin	Hydroxycoumarins	1.368 ± 0.501	LP
Conidendrin	Lignans	1.345 ± 0.300	LP
*p*-Coumaroyl glucose	Hydroxycinnamics	1.331 ± 0.243	LP
Myricetin 3-*O*-arabinoside	Flavonols	1.317 ± 0.423	LP
Arbutin	Other polyphenols	1.309 ± 0.210	LP and CP
Quercetin 3-*O*-xyloside	Flavonols	1.303 ± 0.127	LP
8-Prenylnaringenin	Other flavonoids	1.295 ± 0.418	LP and CP
Ellagic acid arabinoside	Hydroxybenzoics	1.251 ± 0.192	LP
Tetramethylscutellarein	Other flavonoids	1.250 ± 0.357	LP
Kaempferol 7-*O*-glucoside	Flavonols	1.248 ± 0.340	LP
5,6-Dihydroxy-7,8,3′,4′-tetramethoxyflavone	Other flavonoids	1.240 ± 0.327	LP
3-Caffeoylquinic acid	Hydroxycinnamics	1.233 ± 0.077	Meat metabolite
1,5-Diferuloylquinic acid	Hydroxycinnamics	1.229 ± 0.187	LP
5-(3′,5′-dihydroxyphenyl)-gamma-valerolactone 3-*O*-glucuronide	Hydroxyphenylpentanoics	1.218 ± 0.147	Meat metabolite
5-5′-Dehydrodiferulic acid	Hydroxycinnamics	1.197 ± 0.146	CP
Caffeic acid 3-*O*-glucuronide	Hydroxycinnamics	1.188 ± 0.051	MWP
(-)-Epicatechin 3-*O*-gallate	Flavanols	1.182 ± 0.091	Meat metabolite
e-Viniferin	Stilbenes	1.142 ± 0.258	LP
Ellagic acid glucoside	Hydroxybenzoics	1.139 ± 0.198	LP
Luteolin 7-*O*-glucoside	Other flavonoids	1.113 ± 0.116	LP
Ellagic acid	Hydroxybenzoics	1.112 ± 0.267	LP
Piceatannol	Stilbenes	1.086 ± 0.383	MWP
Gallagic acid	Hydroxybenzoics	1.081 ± 0.256	Meat metabolite
Dihydroresveratrol	Stilbenes	1.072 ± 0.234	CP and MWP

Abbreviations: variables’ importance in projection (VIP); orthogonal projections to latent structures discriminant analysis (OPLS-DA).

**Table 3:** Semi-quantitative contents of polyphenols (expressed as mean value, n = 3) in insect-added burgers prior to the in vitro digestion (cooked), together with their changes during in vitro gastrointestinal digestion, considering oral, gastric, and pancreatic post-digestion phases. The % of bioaccessibility value for each digestion phase is reported in round brackets.

Phenolic Equivalents	Burger Sample	Cooked (mg/kg)	Oral Phase (mg/kg)	Gastric Phase (mg/kg)	Pancreatic Phase (mg/kg)
Anthocyanins	CTR	0.174	0.005 (2.9%)	0.048 (27.6%)	0.002 (1.4%)
	CP	0.218	0.006 (2.7%)	0.018 (8.2%)	0.003 (1.4%)
	MWP	0.226	0.002 (0.9%)	0.025 (10.9%)	0.002 (0.7%)
	LP	0.517	0.003 (0.6%)	0.021 (4.1%)	0.002 (0.4%)
Favan-3-ols	CTR	0.849	0.007 (0.8%)	0.048 (5.6%)	0.162 (19.1%)
	CP	1.440	0.005 (0.4%)	0.033 (2.3%)	0.124 (8.6%)
	MWP	2.003	0.008 (0.4%)	0.071 (3.6%)	0.116 (5.8%)
	LP	1.244	0.007 (0.5%)	0.055 (4.4%)	0.148 (11.9%)
Flavones	CTR	0.351	0.001 (0.2%)	0.002 (0.7%)	0.062 (17.8%)
	CP	2.047	<0.001 (0.01%)	0.009 (0.4%)	0.063 (3.1%)
	MWP	0.500	<0.001 (0.1%)	0.009 (1.8%)	0.064 (12.9%)
	LP	2.830	<0.001 (0.01%)	0.002 (0.1%)	0.066 (2.3%)
Flavonols	CTR	0.645	0.004 (0.5%)	0.039 (6%)	0.111 (17.2%)
	CP	0.794	0.002 (0.4%)	0.025 (3.1%)	0.121 (15.3%)
	MWP	0.536	0.003 (0.5%)	0.051 (9.5%)	0.132 (24.6%)
	LP	3.341	0.002 (0.05%)	0.014 (0.4%)	0.160 (4.8%)
Phenolic acids	CTR	28.690	0.652 (2.3%)	2.998 (10.4%)	10.155 (35.4%)
	CP	37.093	0.349 (0.9%)	2.822 (7.6%)	12.078 (32.6%)
	MWP	32.486	0.431 (1.3%)	3.032 (9.3%)	10.197 (31.4%)
	LP	96.415	0.480 (0.5%)	2.417 (2.5%)	10.970 (11.4%)
Other phenolics	CTR	12.823	0.150 (1.2%)	0.654 (5.1%)	1.349 (10.5%)
	CP	21.021	0.197 (0.9%)	1.081 (5.1%)	1.245 (5.9%)
	MWP	15.499	0.194 (1.3%)	1.138 (7.3%)	0.794 (5.1%)
	LP	23.650	0.185 (0.8%)	1.161 (4.9%)	1.778 (7.5%)
Stilbenes	CTR	1.129	0.003 (0.3%)	0.024 (2.1%)	0.014 (1.2%)
	CP	3.024	0.002 (0.1%)	0.023 (0.8%)	0.018 (0.6%)
	MWP	5.498	0.002 (0.04%)	0.016 (0.3%)	0.013 (0.2%)
	LP	3.978	0.003 (0.1%)	0.031 (0.8%)	0.017 (0.4%)

**Table 4:** In vitro antioxidant properties of the different in vitro digested burgers. Values are expressed as mean ± standard deviation (n = 3). Different superscript letters indicate significant differences as resulted from ANOVA (*p* < 0.05; Duncan’s post hoc test).

In Vitro Antioxidant Assay	Digested CTR Burgers	Digested CP Burgers	Digested LP Burgers	Digested MWP Burgers
DPPH (mg TE/mL)	1.60 ± 0.14 ^a^	6.78 ± 0.05 ^c^	6.94 ± 0.17 ^c^	3.02 ± 0.31 ^b^
ABTS (mg TE/mL)	30.59 ± 0.92 ^a^	31.83 ± 0.64 ^ab^	33.19 ± 1.64 ^bc^	35.37 ± 1.40 ^c^
CUPRAC (mg TE/mL)	19.68 ± 0.09 ^a^	23.07 ± 0.28 ^c^	28.82 ± 0.67 ^d^	20.33 ± 0.28 ^b^
FRAP (mg TE/mL)	9.35 ± 0.13 ^a^	14.15 ± 0.11 ^c^	15.91 ± 0.36 ^d^	10.48 ± 0.14 ^b^
Metal chelating (mg EDTAE/mL)	6.07 ± 0.48 ^a^	7.39 ± 0.57 ^b^	6.38 ± 0.39 ^a^	7.82 ± 0.13 ^b^
Phosphomolybdenum (mmol TE/mL)	0.12 ± 0.002 ^a^	0.15 ± 0.001 ^b^	0.15 ± 0.007 ^b^	0.15 ± 0.006 ^b^

Abbreviations: DPPH: 2,2-diphenyl-1-picrylhydrazyl; ABTS: 2,2′-azino-bis(3-ethylbenzothiazoline-6-sulphonic acid); CUPRAC: cupric reducing antioxidant capacity; FRAP: ferric reducing antioxidant power; TE: trolox equivalent; EDTAE: EDTA equivalent.

**Table 5:** Enzyme inhibitory properties of the different in vitro digested burgers. Values are expressed as mean ± standard deviation (n = 3). AChE: acetylcholinesterase; BChE: Butyrylcholinesterase; GALAE: galantamine equivalent; KAE: kojic acid equivalent; ACAE: acarbose equivalent. Different superscript letters indicate significant differences as resulted from ANOVA (*p* < 0.05; Duncan’s post hoc test).

In Vitro Enzymatic Assay	Digested CTR Burgers	Digested CP Burgers	Digested LP Burgers	Digested MWP Burgers
AChE (mg GALAE/mL)	0.09 ± 0.01 ^a^	0.14 ± 0.01 ^b^	0.35 ± 0.01 ^c^	0.10 ± 0.01 ^a^
BChE (mg GALAE/mL)	0.56 ± 0.05 ^a^	0.61 ± 0.04 ^a^	1.26 ± 0.17 ^c^	1.05 ± 0.10 ^b^
Tyrosinase (mg KAE)/mL)	13.44 ± 0.59 ^a^	12.72 ± 1.66 ^a^	12.65 ± 0.84 ^a^	13.51 ± 1.58 ^a^
α-Amylase (mmol ACAE/mL)	0.023 ± 0.001 ^a^	0.024 ± 0.001 ^a^	0.023 ± 0.001 ^a^	0.022 ± 0.001 ^a^
α-Glucosidase (mmol ACAE/mL)	0.117 ± 0.003 ^a^	0.118 ± 0.012 ^a^	0.122 ± 0.012 ^a^	0.117 ± 0.005 ^a^

## Data Availability

The raw data presented in this study are fully available in the article and Supplementary Materials.

## Supplementary Materials

The following supporting information can be downloaded at: https://www.mdpi.com/article/10.3390/antiox13030365/s1, Table S1: the supplementary data file contains the following Excel sheets: (a) UHPLC-HRMS dataset reporting the phenolic compounds annotated in raw insect powders; (b) UHPLC-HRMS dataset reporting the phenolic compounds annotated in cooked insect-containing beef burgers together with the corresponding semi-quantitative values; (c) UHPLC-HRMS dataset reporting the phenolic compounds annotated in digested and cooked insect-containing beef burgers; (d) Pearson’s correlation coefficients between bioactive assays and phenolics at intestinal level; (e) representative UHPLC-HRMS chromatograms of each pooled quality control sample as related to pre- and post-digested samples.
